# Duodenal Web and Pancreas Divisum Causing Pancreatitis in an Adult

**DOI:** 10.1155/1994/58620

**Published:** 1994

**Authors:** J. Kollias, J. Toouli

**Affiliations:** 1 Gastrointestinal Surgical Unit Flinders Medical Centre Bedford Park Adelaide South Australia Australia; 2 Gastrointestinal Surgical Unit Flinders Medical Centre Bedford Park Adelaide South Australia 5042 Australia

## Abstract

Duodenal malformations are the third commonest cause of intestinal obstruction in infants^1^. A spectrum of intrinsic obstructive lesions within the duodenum ranges from atresia to congenital bands^2^. Rarely, duodenal malformations may first present in adulthood. Less than 70 cases of duodenal web presenting in an adult have been reported in the literature. In 10 patients the presentation was associated with pancreatitis. We report a case of congenital duodenal web associated with pancreas divisum which first presented in an adult with the clinical characteristics of recurrent acute pancreatitis.


*HPB Surgery*, 1994, Vol. 7, pp. 231–235
Reprints available directly from the publisher
Photocopying permitted by license only

© 1994 Harwood Academic Publishers GmbH
Printed in the United States of America

### CASE REPORT

### DUODENAL WEB AND PANCREAS DIVISUM CAUSING PANCREATITIS IN AN ADULT

J. KOLLIAS and J. TOOULI
*Gastrointestinal Surgical Unit, Flinders Medical Centre, Bedford Park, Adelaide, South Australia, Australia*

*(Received 12 August 1992)*

Duodenal malformations are the third commonest cause of intestinal obstruction in infants[1]. A spectrum of intrinsic obstructive lesions within the duodenum ranges from atresia to congenital bands[2]. Rarely, duodenal malformations may first present in adulthood. Less than 70 cases of duodenal web presenting in an adult have been reported in the literature. In 10 patients the presentation was associated with pancreatitis. We report a case of congenital duodenal web associated with pancreas divisum which first presented in an adult with the clinical characteristics of recurrent acute pancreatitis.

KEY WORDS: Duodenal web, pancreas division, pancreatitis.

### CASE REPORT

A 29 year old woman presented with an 12 month history of recurrent acute epigastric pain, radiating to the back and associated with vomiting. Each episode would be of 2–12 hours duration and at time be associated with eating a large meal. There was an added history of mild epigastric symptoms resembling reflux over a 5 year period. There was no history of smoking, or excess alcohol consumption, and she did not take regular medications. Physical examination revealed a normally nourished Female with no abnormal physical signs.

An investigation following an episode of pain revealed elevated serum amylase (1,500 $\mu$/ml $n = 100\mu$/ml) normal electrolytes and normal renal and liver function tests. An upper abdominal ultrasound was normal and did not reveal cholelithiasis. Gastrointestinal endoscopy revealed some food residue within the stomach and duodenal cap, some difficulty negotiating the second part of the duodenum but no mucosal abnormality.

In view of the recurrent nature of the pancreatitis an Endoscopic Retrograde Cholangio-Pancreatography (ERCP) was attempted. At this examination, a large food residue was present in the stomach despite a 15 hour fast and the duodenal cap

Address correspondence to: Professor James Toouli, Gastrointestinal Surgical Unit, Flinders Medical Centre, Bedford Park, Adelaide, South Australia, 5042, Australia.

was large and capacious. A small opening was visualised on the medial aspect of the second part of the duodenum resembling the opening of a small duodenal diverticulum. The endoscope could not be negotiated into the opening however the ERCP catheter was introduced through the opening and contrast was injected to reveal normal duodenal mucosa. The papilla was not visualised. An endoscopic diagnosis of duodenal web was made. A barium meal was then performed and this confirmed a large duodenal cap and a 2 mm thick duodenal diaphragm arising from the second part of the duodenum being propelled into the second and third parts giving rise to a "wind sock" appearance (Figure 1). Barium flowed past the diaphragm into the remainder of the duodenum and jejenum. The opening of the diaphragm was confirmed close to the medial attachment of the web. Exclusion of other possible congenital abnormalities by CT and echocardiogram were performed prior to further management.

The patient underwent laparotomy and after mobilising the duodenum, a circumferential indentation of the second part of the duodenal wall was noted. This

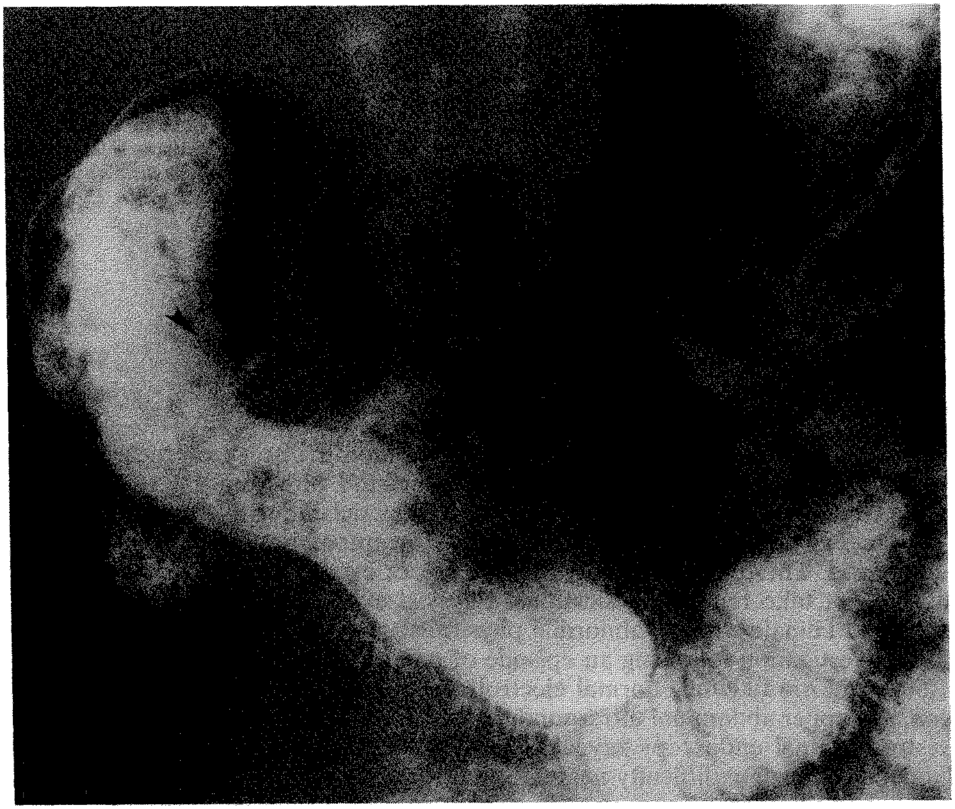

**Figure 1** A barium meal showing the windsock deformity of the duodenal web. The intraduodenal windsock fills the duodenum; the windsock itself is filled with barium. The barium outlines the mucosal lining of the windsock and is seen as quite separate to the barium which lies in the second and third part of the duodenum. The arrow indicates the opening in the web which is situated on the medial aspect of the deformity and close to its attachment to the duodenum. This opening in the web allows for communication between the first and second parts of the duodenum.

indentation marked the insertion site of the web. A longitudinal duodenotomy revealed a 12 cm long duodenal web arising from the entire duodenal circumference and having a 3 mm opening situated near the medial wall (Figure 2). Three separate duct openings were noted on the medial duodenal wall; one opening proximal and two distal to the web. The 3 orifices were sequentially cannulated by a fine catheter and contrast radiography done. In addition the pressures across the duct opening were recorded using a single lumen constantly perfused low compliance catheter. These studies revealed that the orifice proximal to the web drained the body and tail of the pancreas and corresponded to the duct of Santorini.

The two orifices distal to the web drained the bile duct and head of the pancreas respectively and separately, the most distal orifice corresponded to the duct of Wirsung. An abnormal high pressure zone was recorded within the duct of Wirsung but the pressures across the other openings were normal.

The duodenal web was excised leaving a small medial ridge adjacent to the openings of the pancreatobiliary ducts. The excised edge was oversewn in order to achieve haemostasis. The duodenum was then closed transversely. The patients postoperative course was uneventful. Histological examination of the excised specimen confirmed the features of a duodenal web. The submucosal septum was lined by duodenal mucosa on both sides. A thin layer of muscularis mucosa made up the submucosal layer. Two year follow up of the patient revealed no recurrence of symptoms or of pancreatitis.

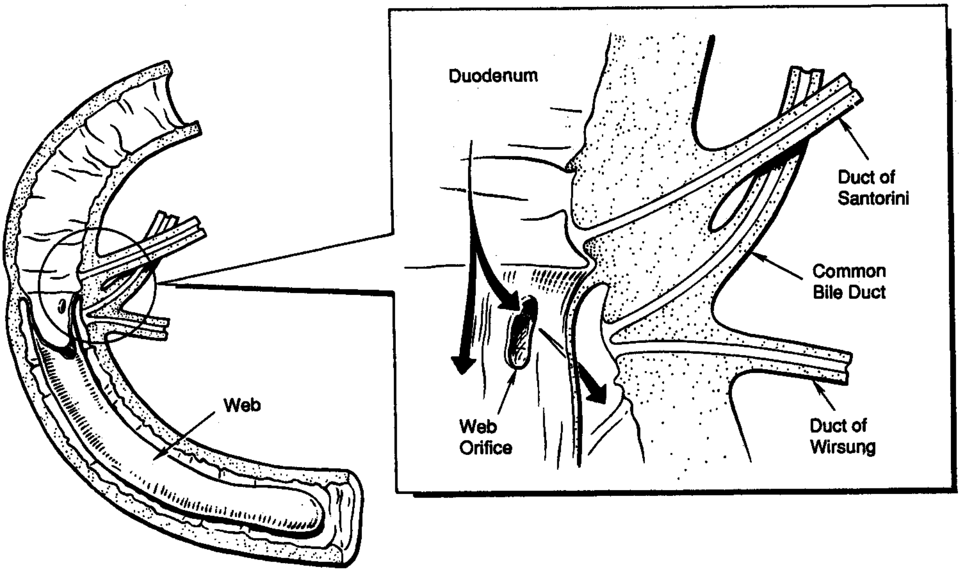

**Figure 2** Schematic representation of the duodenum, the web and their relationships with the common bile duct and pancreatic ducts. The web has a circumferential attachment to the second part of the duodenum and forms a windsock which has been propelled down the second part of the duodenum towards the third part. The opening on the web which allows communication between the first and second parts of the duodenum is situated close to its base on the medial aspect. The insert shows the arrangement of the ducts of Santorini, Wirsung and bile duct and their relationships to the insertion of the web and the web orifice. Note that the attachment of the web separates the duct of Santorini from the common bile duct and duct of Wirsung. The duct of Santorini opens proximal to the base of the web while the other ducts open distal to its attachment.

*Discussion*

Duodenal webs were first reported by Boyd in 1845[3] and are a rare congenital abnormality occurring in 1/9000 live births[4]. They represent part of a spectrum of congenital intrinsic duodenal obstructing lesions that arise due to a defect in recannulisation of the obliterated duodenal lumen during the twelfth week of gestation[1]. The cause of this abnormality is uncertain however it has been postulated to be due to a developmental delay or failure of the foregut and midgut vessels to meet at the level of the ampulla[5]. Duodenal webs may be associated with other congenital abnormalities including Down's Syndrome, congenital heart disease, annular pancreas, gut malrotation and imperforate anus[5,6] but an association with pancreas divisum has not been previously reported. On histology, a duodenal web consists of a double layer of epithelium separated by submucosa and which may or may not have a variable thickness of muscularis. Webs are usually located at or near the bile and pancreatic duct papilla and the openings of these ducts may be found anywhere on the web from the base to close to the web orifice[1,7,8]. Double duodenal webs may occur[9]. In most instances webs are diagnosed in infants due to obstructive symptoms. However occasionally the diagnosis is not made until adulthood. The most common presentation in adults is symptoms of reflux oesophagitis and gastric outlet obstruction. However on rare occasions webs have been associated with pancreatitis. The cause of acute pancreatitis appears to be obstruction of the pancreatic duct either by food residue or distortion of the wall of the web itself[10,20]. Symptoms do not usually appear until the third decade of life, although 20% of patients appear to have had vague abdominal symptoms from childhood[6]. The web may be missed an endoscopy due to their morphological similarity to normal duodenal mucosa. However, the endoscopist should be alerted to the presence of a duodenal obstructing lesion if considerable food residue is present within the duodenal cap and stomach despite adequate periods of fasting by the patient. A side viewing duodenoscope facilitates diagnosis, however a radiological contrast study will usually reveal the diagnosis.

The patient described in this report is unique in that to our knowledge she is the first case of a person with duodenal web associated with the congenital anomaly of pancreas divisum and who presents with recurrent episodes of acute pancreatitis. Manometric recording of the duct orifices revealed pressures consistent with stenosis at the opening of the pancreatic duct draining the head of the pancreas (duct of Wirsung) and not at the other orifices. Stenosis of the opening of the duct of Santorini in pancreas divisum is thought to be associated with recurrent episodes of pancreatitis. It is thought that stenosis of the Duct of Wirsung also may be associated with pancreatitis. Another possibility for the pathogenesis of pancreatitis in this patient might be distortion of the pancreatic duct opening by food being held up proximal to the web. The distortion might effect either the duct of Santorini (proximal to the web) or the duct of Wirsung (distal to the web).

Web excision and transverse duodenoplasty is the surgical treatment of choice[2,4,6,10,11,12-20], but cases of endoscopic snare excision and radial web incision using a papillotome or laser have been described[14,21,22]. Such an approach is potentially hazardous as the ducts may be injured in patients who may have anomalous drainage of the pancreatic and biliary ducts near or within the web. It is recommended that if the endoscopic form of therapy is to be considered ERCP is mandatory in order to delineate these ducts and avoid inadvertent injury[14,16]. If, as in our case the openings cannot be visualised by ERCP, duodenotomy and web excision is recommended. The limited experience of patients treated by open surgery indicates excellent long term results and relief of symptoms.

### *References*

1. Boyden, E.A., Cope, J.A. and Bill, A.H. (1967) Anatomy and Embryology of congenital intrinsic obstruction of the duodenum. *Am.J.Surg.*, **114**, 190–202
2. Kazmers, N. (1966) Duodenal diaphragm in the adult. *A.M.J.Gastroenterol.*, **45**, 342–347
3. Boyd, R. (1845) Description of a malformation of the duodenum. *Lancet*, **1**, 648
4. Cooperman, A.M., Adachi, M., Rankin, F.B. and Sivak, M. (1975) Congenital duodenal diaphgragm in adults. *Ann.Surg.*, **182**, 739--742
5. Davey, R.B. (1980) Congenital intrinsic duodenal obstruction: A comparative review of associated anomalies. *Aust.Paediatr.J.*, **16**, 274–278
6. Economides, N.G., McBurney, R.P. and Hamilton, F.H. (1977) Intraluminal duodenal diverticulum in the adult. *Ann.Surg.*, **185**, 147–152
7. Zatzkin, H.R., Macy, J.J. and Kveton, F.W. (1959) Intraluminal duodenal diverticulum: Report of a case. *AJR*, **82**, 1036–1037
8. Richardson, W.R. and Martin, L.W. (1969) Pitfalls in the surgical management of the incomplete duodenophr. *J.Pediatr.Surg.*, **4**, 303–312
9. Reiner, R.G., A.L.P.M.H., O'Brien, J.A. and Jones, G.H. (1978) Double Duodenal Diaphragm: Report of a case. *Aust.N.Z.J.Surg.*, **48**, 310–313
10. Sheridan, R.L., Shay, S.S. and D'Avis, J.C. (1986) *Am.J.Gastroenterol.*, **81**, 718–720
11. Suarez, L.A. and Bolden, E.I. (1978) *Curr.Surg.*, **35**, 366–369
12. Kazmers, N. (1966) *Amer.J.Gastroenter.*, **45**, 342
13. Ferraris, V.A. and McPhail, J.F. (1984) *Surg.Gyn.Obs.*, **158**, 461
14. Killebrew, L. and Kukora, J.S. (1983) *Arch.Surg.*, **118**, 875
15. Soreide, J.A., Seime, S. and Soreide, D. (1988) *Amer.J.Gastroenter.*, **83**, 988
16. Cooperman, A.V., Adachi, M., Rankin, G.B. and Sivak, M. (1975) *Ann.Surg.*, **182**, 739
17. Anderson, T.H. and Deitel, M. (1984) *Can.J.Surg.*, **27**, 365
18. Smiley, K., Perry, M. and McClelland, R. (1967) *Ann.Surg.*, **165**, 362
19. Adams, D.B.
20. Howard, J.M., Wynn, O.B., Lenhart, F.M. andChandnani, P.C. (1986) *Amer.J.Surg.*, **151**, 505
21. Jex, R.K. and Hughes, R.W. (1986) *Gastrointest.Endosc.*, **32**, 416
22. Al-Kawas, F.H. (1988) *Gastrointest.Endosc.*, **34**, 349

*(Accepted by S.Bengmark 21 January 1993)*

